# Antibodies against carbamylated proteins and cyclic citrullinated peptides in systemic lupus erythematosus: results from two well-defined European cohorts

**DOI:** 10.1186/s13075-016-1192-x

**Published:** 2016-12-03

**Authors:** Michael Ziegelasch, Myrthe A. M. van Delft, Philip Wallin, Thomas Skogh, César Magro-Checa, Gerda M. Steup-Beekman, Leendert A. Trouw, Alf Kastbom, Christopher Sjöwall

**Affiliations:** 1 Rheumatology/AIR, Department of Clinical and Experimental Medicine, Linköping University, Linköping, Sweden; 2 Department of Rheumatology, Leiden University Medical Center, C1-R, LUMC, PO Box 9600, Leiden, 2300 RC The Netherlands; 3 Rheumatology Unit, University Hospital, Linköping, SE-581 85 Sweden

**Keywords:** Anti-CarP, Anti-CCP, Rheumatoid arthritis, Rheumatoid factor, Systemic lupus erythematosus, Ultrasonography

## Abstract

**Background:**

Articular manifestations are common in systemic lupus erythematosus (SLE) whereas erosive disease is not. Antibodies to cyclic citrullinated peptide (anti-CCP) are citrulline-dependent in rheumatoid arthritis (RA), whereas the opposite is suggested in SLE, as reactivity with cyclic arginine peptide (CAP) is typically present. Antibodies targeting carbamylated proteins (anti-CarP) may occur in anti-CCP/rheumatoid factor (RF)-negative cases long before clinical onset of RA. We analysed these antibody specificities in sera from European patients with SLE in relation to phenotypes, smoking habits and imaging data.

**Methods:**

Cases of SLE (n = 441) from Linköping, Sweden, and Leiden, the Netherlands, were classified according to American College of Rheumatology (ACR) and/or Systemic Lupus Erythematosus International Collaborating Clinics (SLICC) criteria. IgG anti-CCP, anti-CAP and anti-CarP were analysed by immunoassays. Radiographic data from 102 Swedish patients were available.

**Results:**

There were 16 Linköping (6.8%) and 11 Leiden patients (5.4%) who were anti-CCP-positive, of whom approximately one third were citrulline-dependent: 40/441 (9.1%) were anti-CarP-positive, and 33% of the anti-CarP-positive patients were identified as anti-CCP-positive. No associations were found comparing anti-CCP or anti-CarP with ACR-defined phenotypes, immunologic abnormalities or smoking habits. Radiographically confirmed erosions were found in 10 patients, and were significantly associated with anti-CCP, anti-CarP and RF. Musculoskeletal ultrasonography scores were higher in anti-CCP-positive compared to anti-CCP-negative patients.

**Conclusions:**

In the hitherto largest anti-CarP study in SLE, we demonstrate that anti-CarP is more prevalent than anti-CCP and that the overlap is limited. We obtained some evidence that both autoantibodies seem to be associated with erosivity. Similar pathogenetic mechanisms to those seen in RA may be relevant in a subgroup of SLE cases with a phenotype dominated by arthritis.

## Background

In clinical practice, the diagnosis of systemic lupus erythematosus (SLE) is often based on the involvement of at least two organ systems combined with a diversity of immunological abnormalities [1]. The presence of antinuclear antibodies (ANA) and reduced levels of circulating complement proteins are typical immunological abnormalities in SLE, and both of these are included in the most recent proposal of classification criteria [2]. Over the years, several attempts to link specific autoantibodies to certain clinical phenotypes of SLE have been made. For instance, antibodies against double-stranded DNA (dsDNA) and C1q are commonly found in lupus nephritis [3, 4], anti-Ro/SSA antibodies often coincide with lupus-related rash and photosensitivity [5], and anti-phospholipid antibodies are frequently found in patients with SLE who have thromboembolic events [6].

Detection of antibodies against cyclic citrullinated peptide (anti-CCP) is an important diagnostic and prognostic tool in arthritis, as it is highly specific for rheumatoid arthritis (RA) and predictive of erosive disease. While a positive anti-CCP test in RA is typically citrulline-dependent [7], it has been suggested that anti-CCP in other conditions is generally not, and thus, also reacts with the corresponding cyclic arginine peptide (anti-CAP) [8, 9]. During the last years, it has been repeatedly shown that antibodies targeting carbamylated proteins (anti-CarP) may occur in anti-CCP/rheumatoid factor (RF) negative cases [10–12]. Like RF and anti-CCP, anti-CarP antibodies can also be detected many years before the onset of RA [13–15].

The process of carbamylation is mediated by a chemical reaction of cyanate with mainly lysine residues in proteins [16]. Cyanate is present in the body in equilibrium with urea. Inflammation, smoking and renal failure have been reported to increase the non-enzymatic post-translational modification in which cyanate binds to molecules containing primary amine or thiol groups and forms carbamyl groups [16]. Carbamylation of proteins can lead to the loss of tolerance with formation of antibodies directed against carbamylated proteins (anti-CarP antibodies) in susceptible individuals [10–12].

Over the last years, this novel group of autoantibodies has been intensively studied in patients before the onset of clinical RA symptoms and in patients with established RA, in relation to prognostic factors such as anti-CCP/RF and to disease outcomes (i.e. radiological progression) [10, 17, 18]. Anti-CarP has also been recently reported in primary Sjögren’s syndrome, and found to be strongly associated with increased focal lymphocyte infiltration, formation of ectopic germinal centre-like structures, and to the degree of affected salivary gland function [19]. To our knowledge, only two previous small studies have addressed the occurrence of anti-CarP antibodies in SLE. Thus, Scinocca et al. reported the occurrence of anti-CarP (homocitrullinated fibrinogen) antibodies in 49% of 81 patients with RA, but in none of 37 patients with SLE, 37 patients with psoriatic arthritis, or 27 healthy controls [20]. In the study by López-Hoyos et al., 48% (16/33) of anti-CCP/RF-negative patients with elderly-onset RA were judged seropositive for anti-CarP compared to 39% (48/124) of patients with polymyalgia rheumatica, and 11% (4/37) of patients with SLE [21].

Although articular involvement is common in SLE (at least 80%) and often constitutes the presenting symptom, it has historically received limited attention [22, 23]. A plausible reason hereto is that arthritis causes significant problems to the patient at disease onset, but often responds rather well to treatment with glucocorticoids, hydroxychloroquine and methotrexate [22]. Development of deformities is occasionally seen, but the vast majority of patients with SLE do not develop radiographically evident erosions [22]. Accordingly, several investigators have estimated the frequency of erosive arthritis in SLE to be 2–5%, but the finding of erosions on radiography often complicates the distinction between SLE and RA [22, 24]. As a result, the concept of “rhupus” was introduced to describe patients who simultaneously fulfil classification criteria for both conditions [25, 26]. It is still a matter of debate as to whether this group represents a true overlap of RA and SLE, rather than a subset of SLE [27, 28].

Hitherto, only a few studies of lupus arthritis based on imaging modalities other than conventional radiography have been published. A limited number of studies have evaluated magnetic resonance imaging (MRI) in the assessment of patients with articular involvement in SLE [29–32]. Recently, Ball and colleagues demonstrated that MRI is highly sensitive in identifying erosions, synovitis and bone oedema, independently of anti-CCP and RF antibody status [32]. In SLE, there are some reports based on musculoskeletal ultrasonography (US), but so far this modality has seldom been used in clinical routine practice [31, 33].

The primary aim of this study was to describe the presence of citrulline-dependent anti-CCP and anti-CarP antibodies in 441 well-characterized patients within two European SLE cohorts. Second, in the Swedish dataset, we aimed to investigate the associations between autoantibody status and articular involvement as defined by classification criteria, conventional radiographic data and evaluation with musculoskeletal US.

## Methods

### Discovery cohort

Patients diagnosed with SLE (n = 236) were included in the discovery cohort. All patients took part in the prospective, structured follow-up programme “KLURING” (the Swedish acronym for *Clinical Lupus Register In Northeastern Gothia*) at the rheumatology outpatient clinic, University Hospital, Linköping, Sweden. The patient material has previously been described in detail [3, 34]. Patients were recruited consecutively and classified as having SLE according to the 1982 American College of Rheumatology (ACR) criteria and/or the 2012 Systemic Lupus International Collaborating Clinics classification criteria (SLICC)-12 [2, 35]. Most patients represented prevalent cases (83%), but 41 patients (17%) had newly diagnosed disease at inclusion. Information on smoking habits (current/former/never) and activity limitations defined according to the validated Swedish version of the health assessment questionnaire (HAQ) was recorded at the time of blood sampling [36]. Further patient characteristics are summarized in Table 1.

**Table 1:** Clinical and immunological features of the 441 included patients

Patient characteristics	Discovery cohort (n = 236)	Replication cohort (n = 205)	Mann–Whitney *U* test or chi-square test, *p* value
Background variables			
Female, % (*n*)	88% (207)	89% (183)	N.S.
Age, years, median (range)	54 (19–94)	42 (13–80)	<0.001
Disease duration, years, median (range)	15 (0–52)	8 (0–32)	<0.001
Caucasian ethnicity, % (*n*)	93% (219)	69% (142)	<0.001
Conventional radiology available, % (*n*)	43% (102)	N.A.	
Erosions on x-ray, % (*n*)	4.2% (10)^a^	N.A.	
HAQ score (median, range)	0.13 (0-3)	N.A.	
Ever smoker (former or current), % (*n*)	45% (107)	43% (88)	N.S.
Meeting ACR-82, % (*n*)	85% (201)	91% (188)	0.038
Meeting SLICC-12, % (*n*)	99% (233)	99% (203)	N.S.
Clinical phenotypes (SLICC-12 definitions on criteria 1 − 11), % (*n*)			
1) Acute cutaneous lupus	45% (106)	53% (108)	N.S.
2) Chronic cutaneous lupus	15% (36)	18% (36)	N.S.
3) Oral ulcers	12% (29)	32% (66)	<0.001
4) Non-scarring alopecia	22% (51)	19% (39)	N.S.
5) Synovitis	76% (180)	70% (143)	N.S.
6) Serositis	37% (87)	23% (48)	0.003
Pleuritis	35% (83)	18% (37)	<0.001
Pericarditis	14% (34)	14% (28)	N.S.
7) Renal	28% (66)	26% (53)	N.S.
8) Neurologic	11% (26)	22% (44)	0.004
Seizures	4.2% (10)	5.4% (11)	N.S.
Psychosis	1.7% (4)	4.4% (9)	N.S.
Mononeuritis multiplex	0.4% (1)	0% (0)	N.S.
Myelitis	0.4% (1)	2.9% (6)	N.S.
Peripheral or cranial neuropathy	5.1% (12)	8.3% (17)	N.S.
Acute confusional state	0.8% (2)	2% (4)	N.S.
9) Haemolytic anaemia	4.7% (11)	5.9% (12)	N.S.
10) Leukopenia and/or lymphopenia	52% (122)	30% (62)	<0.001
11) Thrombocytopenia	12% (28)	18% (37)	N.S.
Raynaud	26% (61)	40% (82)	0.002
Interstitial lung disease	3.5% (8)	3.4% (7)	N.S.
Immunological features (SLICC-12 definitions on criteria 1 to 6), % (*n*)			
1) Antinuclear antibody (ANA)	100% (236)	100% (205)	N.S.
2) Anti-dsDNA antibody (anti-dsDNA)	50% (118)	56% (115)	N.S.
3) Anti-Smith antibody (anti-Sm)	8.1% (19)	10% (21)	N.S.
4) Antiphospholipid antibody	59% (139)	44% (90)	0.002
Lupus anticoagulant	35% (69)^b^	32% (65)	N.S.
Anti-cardiolipin antibody	34% (80)	24% (49)	0.027
Anti-β2-glycoprotein I antibody	26% (62)	18% (27)^c^	N.S.
5) Low complement	53% (124)	50% (102)	N.S.
6) Direct Coombs test	56% (59)^d^	23% (38)^e^	<0.001
Anti-small nuclear ribonucleoprotein antibody (anti-snRNP)	37% (88)	17% (35)	<0.001
Anti-Ro/Sjögren’s syndrome A antibody (SSA)	37% (88)	42% (87)	N.S.
Anti-La/Sjögren’s syndrome B antibody (SSB)	28% (66)	13% (27)	<0.001
Anti-cyclic citrulline peptide antibody (anti-CCP)^f^	6.8% (16)	5.4% (11)	N.S.
Anti-carbamylated protein antibody (anti-CarP)	9.8% (23)	8.3% (17)	N.S.
Rheumatoid factor^f^	25% (26)^d^	15% (31)	0.046

^a^Calculated in 236 patients. ^b^Data available on 195 patients. ^c^Data available on 148 patients. ^d^Data available on 107 patients. ^e^Data available on 164 patients. ^f^Not performed with identical assays. *HAQ* health assessment questionnaire, *ACR* American College of Rheumatology, *SLICC* Systemic Lupus Erythematosus International Collaborating Clinics, *ANA* antinuclear antibodies, *dsDNA* double-stranded DNA, *N.A.* not applicable, *N.S.* not significant

It should be emphasized that the definition of arthritis slightly differs between the ACR-82 and SLICC-12 criteria. Whereas ACR-82 requires “non-erosive arthritis involving ≥2 peripheral joints, characterized by tenderness, swelling, or effusion”, the presence of “synovitis involving 2 or more joints, characterized by swelling or effusion OR tenderness in 2 or more joints and at least 30 minutes of morning stiffness” is demanded to meet the SLICC-12 arthritis criterion.

### Replication cohort

A total of 205 consecutive patients with SLE were included in the replication cohort. All patients were selected from the Leiden NPSLE clinic, at the rheumatology department, Leiden University Medical Center (LUMC), the Netherlands. The LUMC serves as a national referral centre for patients with SLE suspected of having neuropsychiatric involvement. All patients included were admitted for one day and underwent a complete examination that has previously been described in detail [37]. All patients were classified as having SLE according to the 1997 updated ACR-82 and/or the SLICC-12 criteria [2, 38]. Data on gender, smoking status and ethnicity were retrospectively recorded. Detailed patient characteristics relevant to the present study are summarized in Table 1.

The following definitions of smoking habits were used in both cohorts [39]. “Former smoker” means to have ever been a regular smoker; occasional smokers were not included. “Ever smoker” constitutes the sum of “former smoker” and “current smoker”.

### Laboratory analyses

In the discovery cohort, IgG-class anti-CCP and anti-CAP antibodies were analysed by enzyme-linked immunosorbent assays (CCPlus and anti-CAP respectively, Euro-Diagnostica, Malmö, Sweden). Levels ≥25 units/mL defined a positive test as suggested by the manufacturer. Citrulline dependency was defined as a higher antibody level obtained in the anti-CCP test than in the anti-CAP test. RF was detected by nephelometry at the clinical immunology department, University Hospital, Linköping, Sweden.

For the replication cohort, anti-CCP and anti-CAP antibodies were analysed using an in-house CCP2 assay with a defined cutoff ≥25 units/mL [40]. Healthy controls (n = 193) who were living in the Leiden area, were included for comparison. Briefly, biotinylated CCP2 citrulline and corresponding arginine control peptide were coupled to streptavidin-coated enzyme-linked immunosorbent assay (ELISA) plates. Serum samples were diluted 1:50 and bound IgG was detected using rabbit anti-human IgG horseradish peroxidase (HRP) (DAKO, Glostrup, Denmark).

Analysis of IgG anti-CarP antibody in both cohorts was performed in Leiden, using a previously described immunoassay detecting antibodies against carbamylated fetal calf serum proteins [10]. Other laboratory analyses, including IgM RF quantified by fluoroenzyme immunoassay, were performed at the routine diagnostic laboratory of the Leiden University Medical Center, Leiden, the Netherlands.

### Radiology

Through meticulous chart review of each patient, we identified all conventional radiographs of the hands, wrists and/or feet, obtained at the University Hospital, Linköping (discovery cohort). The results were scrutinized by an experienced rheumatologist (MZ) who was blinded to the patients’ antibody status. In cases where erosions had been identified by the radiologist and in all cases with indistinct findings, the radiographs were finally evaluated by MZ to determine whether erosions were present.

### Musculoskeletal US

In the discovery cohort, all anti-CCP-positive cases plus just as many anti-CCP-negative cases, matched according to sex, age, disease duration and present prednisolone dose, were systematically investigated with musculoskeletal US by an experienced examiner (MZ) blinded to the patients’ antibody profiles and conventional radiography results. Characteristics of the 32 patients, representing a subgroup of the discovery cohort, are shown in Table 2.

**Table 2:** Characteristics of the 32 discovery cohort patients evaluated with musculoskeletal ultrasonography

	Anti-CCP-positive (n = 16)	Anti-CCP-negative (n = 16)	*P* value
Age, years, median	58	58	N.S.
Disease duration, years, median	10.5	10.5	N.S.
Female, number	14	13	N.S.
Fulfilled ACR-82 criteria number, median	4	4	N.S.
Meeting arthritis criterion according to ACR-82, number	13	14	N.S.
Meeting nephritis criterion according to ACR-82, number	4	2	N.S.
Conventional radiographs available (hands/wrists/feet)	16	16	N.S.
Erosions on radiography, number of individuals	4	0	N.S.
HAQ score, median	0.5	0.19	N.S.
Ever smoker, number of individuals	9	3	N.S.
Rheumatoid factor positive, number of individuals	6	1	N.S.
anti-CarP antibody level, median	275	102	0.04
anti-CarP positive, number of individuals	6	1	N.S.
Daily dosage of prednisolone, median	2.5	2.5	N.S.

*ACR* American College of Rheumatology, *HAQ* health assessment questionnaire, *anti-CarP* antibodies targeting carbamylated proteins, *N.S*. not significant

US examination was performed using the LOGIQ-E9 (GE Healthcare, Milwaukee, WI, USA) with a linear scanner 6–15 MHz. All patients were examined with the same settings for both B-mode (grey scale) and power Doppler (PD). The protocol included dorsal assessments of the following 36 joints: bilateral radiocarpal, intercarpal, distal radioulnar, metacarpophalangeal (MCP) I–V, interphalangeal (IP) thumb joints, proximal interphalangeal (PIP II-V), and metatarsophalangeal (MTP) joint I-V. In addition, six tendons (extensor carpi ulnaris in the wrist, tibialis posterior and flexor digitorum longus in the feet) were evaluated. To grade synovitis, we used the scoring system of Szkudlarek et al. in which synovial hypertrophy is graded 0–3 (0 = no thickening, 3 = synovial thickening bulging over the tops of the periarticular bones and extension over the diaphysis of at least one side) and perfusion 0–3 (0 = no Doppler signals in the synovium, 3 = confluent Doppler signals in more than one half of the synovium) [41, 42].

### Statistics

Potential associations between antibody status and clinical characteristics were tested by Fisher’s exact test for categorical variables and by the Mann-Whitney *U* test for numerical variables. The Mann-Whitney *U* test or chi-square test was used to evaluate differences between the cohorts. Statistical analyses were performed using SPSS v23.

For analyses where we had prior hypotheses, a significance level of 5% was regarded as statistically significant (two-sided *p* values <0.05). For all other tests performed in a more exploratory manner, the exact *p* values (if *p* was <0.05) are reported as the reference.

## Results

### Comparison between cohorts

As shown in Table 1, the size of the two cohorts was similar, whereas in some instances there were significant differences in the clinical phenotypes according to the classification criteria that were fulfilled (oral ulcers, serositis, neurological involvement, Raynaud). Significantly more patients in the discovery cohort were older, had longer disease duration, and were Caucasian than in the replication cohort. In addition, laboratory criteria such as the presence of leukopenia/lymphocytopenia, antiphospholipid antibody, anti-snRNP antibody, anti-La/SSB antibody, RF and the direct Coombs test differed between the cohorts.

### Presence of anti-CCP/CAP/CarP antibodies in SLE

**Fig. 1:**
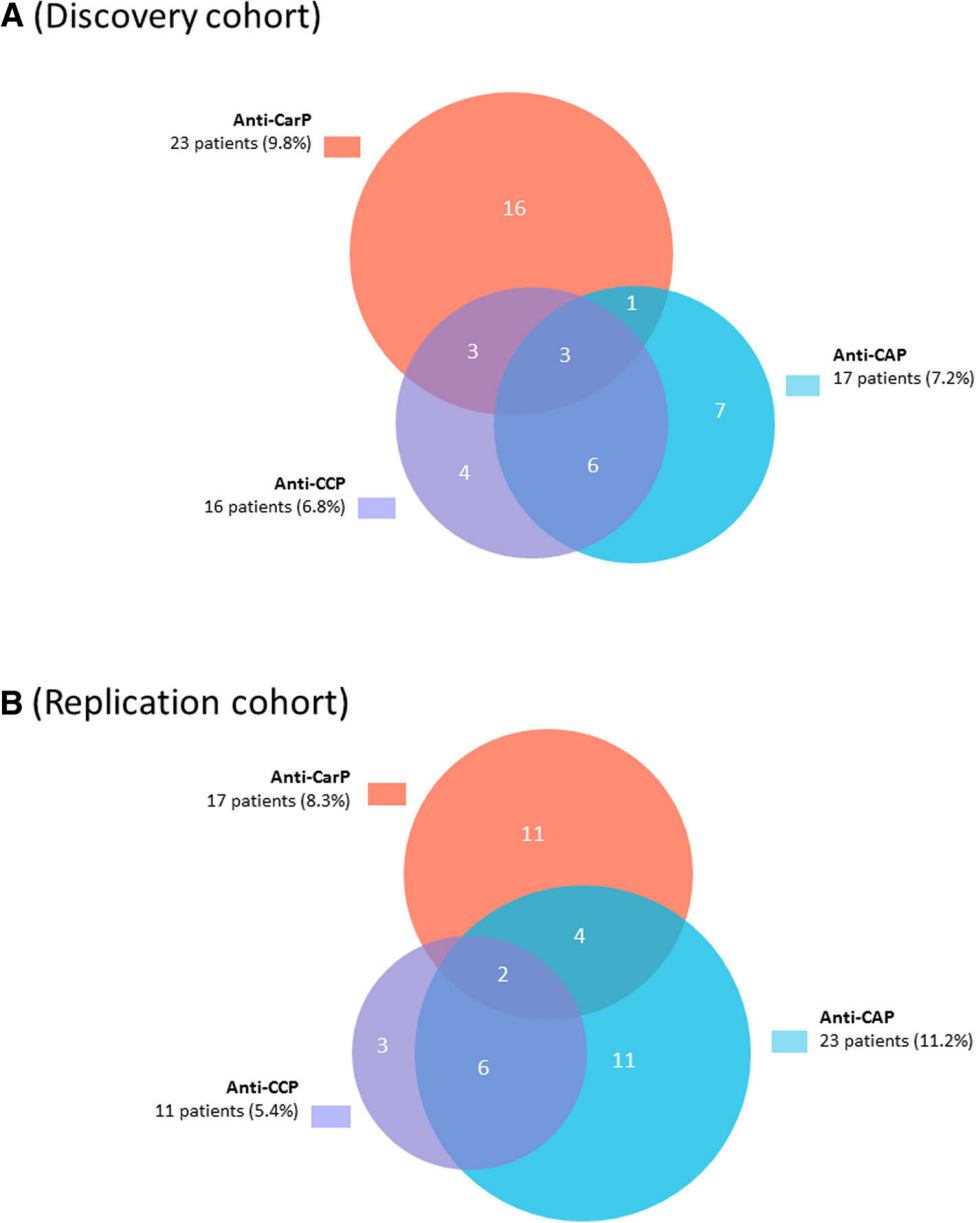
**a**-**b** Distribution of anti-carbamylated protein (*anti-CarP*)-positive, anti-cyclic citrullinated peptide (*anti-CCP*)-positive and anti-cyclic arginine peptide (*anti-CAP*)-positive patients in the discovery cohort (n = 236) (**a**), and in the replication cohort (n = 205) (**b**)

In the discovery cohort, 16 patients (6.8%) were anti-CCP-positive, 9 (56%) of whom were also anti-CAP-positive using Euro-Diagnostica kits; however, only one of the 9 anti-CCP/anti-CAP-positive patients had a higher antibody level for anti-CAP than for anti-CCP in the assays: 4 of the 7 patients with a positive citrulline-dependent anti-CCP test had a history of biopsy-proven lupus nephritis. There were 23 anti-CarP-positive patients (9.8%); only 6 (26%) of the anti-CarP-positive patients were identified as anti-CCP-positive (Fig. 1a).

In the replication cohort, 11 patients (5.4%) were anti-CCP-positive, of whom 8 (73%) were also positive for anti-CAP using in-house assays; in 4 of the 8 anti-CCP/anti-CAP-positive patients, there was a higher optical density signal for anti-CAP than for anti-CCP. There were 17 anti-CarP-positive patients (8.3%), of whom 2 (12%) were also identified as anti-CCP-positive (Fig. 1b). A limited number of samples from the discovery cohort were also analysed with the anti-CCP in-house assay in Leiden. The agreement between the assays was fair (*rho* = 0.75; concordance 90%).

### Associations between anti-CCP, anti-CarP, or RF and clinical or other laboratory features

In the discovery cohort, neither anti-CCP nor anti-CarP were associated with arthritis by classification according to physical examination. However, the presence of anti-CarP was associated with neurological involvement as defined by SLICC-12 (*p* = 0.028); this association was particularly driven by the presence of cranial/peripheral neuropathy (*p* = 0.021). On the contrary, anti-CarP was less common among patients with a positive lupus anticoagulant test (*p* = 0.012). Positive anti-CCP tests were inversely related to anti-La/SSB (*p* = 0.007). Smoking habits and HAQ score were not associated with anti-CCP, anti-CarP or RF.

In the replication cohort, the presence of anti-CarP was not significantly associated with neurological involvement as defined by SLICC-12 (*p* = 0.126); and there was no association with specific neurological manifestations included in the SLICC-12 criteria. Anti-CarP was not associated with any other SLICC-12 definition or SLE autoantibody. On the contrary, anti-CCP was more common among patients with positive anti-Sm (*p* = 0.017). There was no association between anti-CCP positivity and anti-La/SSB. Smoking habits were not associated with anti-CCP, anti-CarP or RF.

### Associations between anti-CCP, anti-CarP or RF, and radiography

In the Swedish cohort, conventional radiographs of the hands, wrists and/or feet were available in 102 patients (43%), and erosions (mostly demonstrated in the intercarpal, MCP and MTP joints) were identified in 10 patients. This corresponds to 4.2% calculated for the entire Swedish cohort, and to 9.8% calculated strictly for patients with radiographs available. Radiological erosions were significantly associated with a positive anti-CCP (*p* < 0.05), anti-CarP (*p* < 0.05) or RF (*p* < 0.05) test, respectively.

All patients with a positive anti-CCP test underwent radiography of the hands, wrists and feet. Thus, patients with radiographs available were more often anti-CCP-positive (16/102; 16%) as compared to patients without radiographs (0/134; *p* < 0.001). Similarly, “ever”’ smokers were significantly overrepresented among patients with radiographs (55/102; 54%) compared to those without (52/134; 39%; *p* = 0.025). There were no significant differences in age, sex or fulfilment of the SLICC-12 arthritis criterion. Neither did the occurrence of anti-CarP, anti-CAP or RF significantly differ according to the availability of radiographic data.

### Associations between anti-CCP, anti-CarP or RF, and musculoskeletal US

**Fig. 2:**
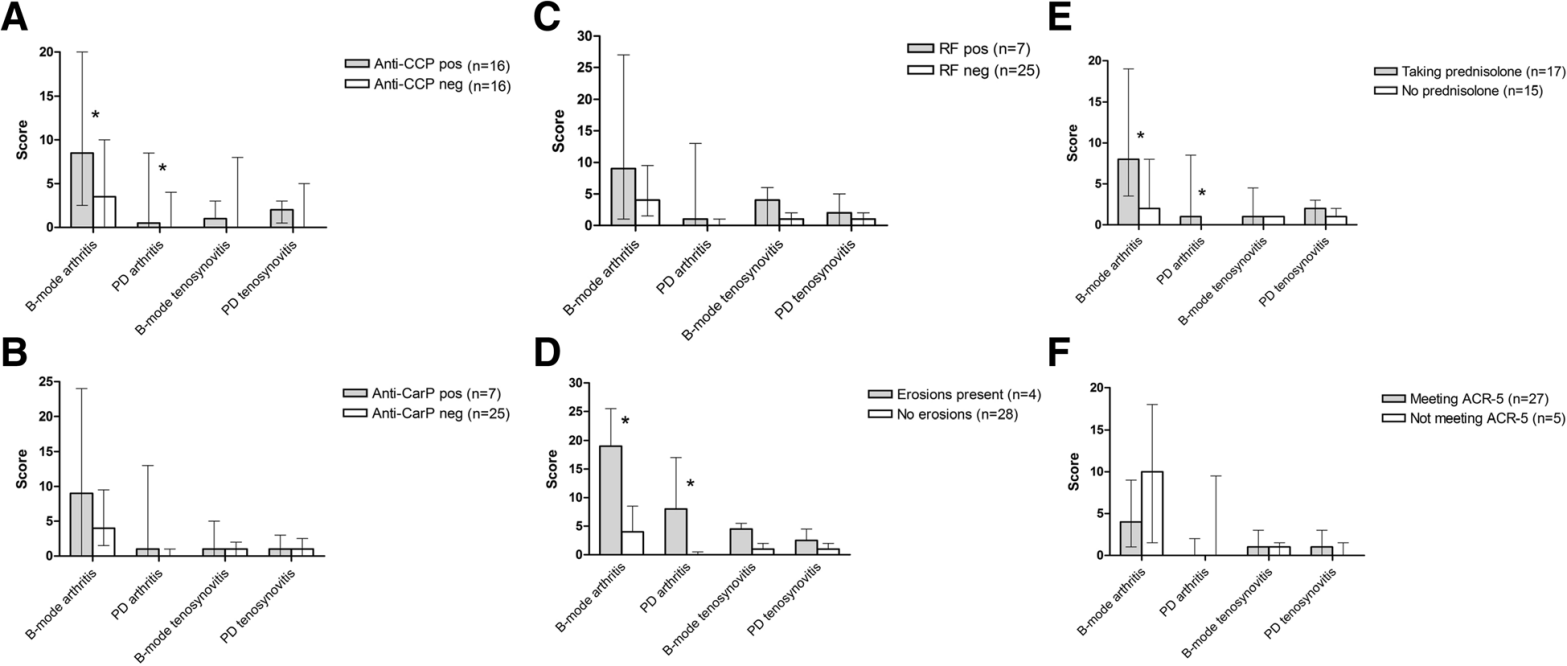
**a**-**f** Standardized musculoskeletal ultrasonography grading of arthritis and tenosynovitis with grey scale (B-mode) and power Doppler (*PD*) in 32 patients divided according to the presence of anti-cyclic citrullinated peptide (*anti-CCP*) (**a**), anti-carbamylated protein antibody (*anti-CarP*) (**b**), rheumatoid factor (**c**), radiographically confirmed erosions (**d**), daily intake of prednisolone (**e**), and with regard to the fulfilment of the 1982 American College of Rheumatology (*ACR*) classification criterion 5 (arthritis) (**f**) [35]. Median and interquartile range are illustrated. * *p* < 0.05

In anti-CCP-positive patients, US examination of the joints and tendons resulted in significantly higher arthritis scores, but not tenosynovitis scores (Fig. 2a). There were similar trends for anti-CarP and RF, but these were not statistically significant (Fig. 2b–c). Patients with radiological erosions had significantly higher arthritis scores, but not tenosynovitis scores (Fig. 2d). Individuals with ongoing prednisolone medication had higher arthritis, but not tenosynovitis scores (Fig. 2e), whereas fulfilment of the ACR criterion for arthritis did not separate the groups (Fig. 2f).

### Associations between anti-CCP or anti-CarP, and the fulfilment of RA classification

**Fig. 3:**
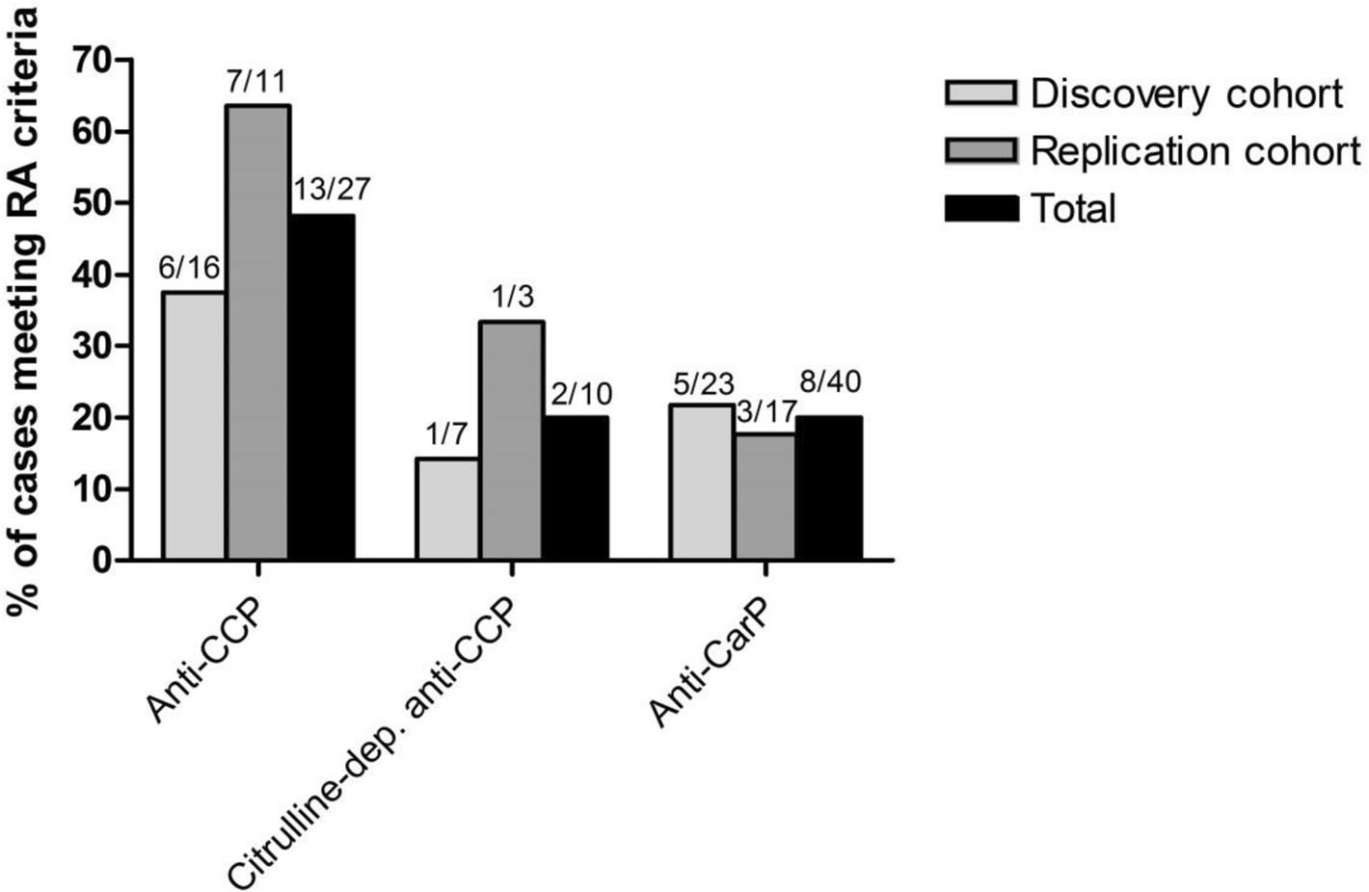
Percentage of patients who, besides being classified as having systemic lupus erythematosus (SLE), also fulfilled the 2010 American College of Rheumatology/European League Against Rheumatism classification criteria for rheumatoid arthritis (*RA*) [43] provided they were: (1) anti-cyclic citrullinated peptide (*anti-CCP*) antibody-positive, (2) identified as citrulline-dependent anti-CCP antibody-positive (i.e. higher anti-CCP than cyclic arginine peptide (*anti-CAP*) antibody level), and (3) anti-carbamylated protein (*anti-CarP*) antibody-positive

Besides meeting the classification criteria for SLE, we evaluated to what extent patients with a positive anti-CCP and/or anti-CarP antibody test simultaneously fulfilled the 2010 ACR/European League Against Rheumatism (EULAR) criteria for RA [43]. As illustrated (Fig. 3), the likelihood of meeting the RA criteria was higher with a positive anti-CCP compared to a positive anti-CarP antibody test. However, when taking citrulline dependency into account for the anti-CCP test this difference was less pronounced, particularly in the discovery cohort.

## Discussion

This is hitherto the largest evaluation of anti-CarP antibodies in SLE, and the first study on anti-CarP in a European SLE population. Herein, we demonstrated similar frequencies of anti-CarP-positive SLE patients in the two cohorts (9.8% vs*.* 8.3%) and that the overlap with anti-CCP antibodies is limited. Our findings are in line with what has been reported by López-Hoyos et al., but clearly higher than observed by Scinocca and co-workers [20, 21]. The latter may be explained by a difference in the antigen used for the detection of anti-CarP antibodies (fibrinogen vs. fetal calf serum). Furthermore, we found significant associations between all three RA-associated antibodies (anti-CCP, anti-CarP and RF) and radiographically confirmed erosions in the Swedish dataset. Based on the results, we hypothesize that pathogenetic mechanisms could be similar in RA and in a small group of patients with SLE with a clinical phenotype dominated by arthritis [44]. Interestingly though, 60% of the patients with radiology proven erosions were not identified by any of the antibodies.

Articular manifestations affect a majority of patients with SLE, at least at some time during the disease course (73% in the present study). However, only a minority of the patients with SLE who have an arthritic phenotype simultaneously meet RA classification criteria [24, 25, 31]. The presence of anti-CCP antibodies is considered highly specific for RA, but can also be found in other conditions, including SLE, where frequencies from 2–17% have been described [9, 32, 45–51]. Whether or not there is a true association between a positive anti-CCP test and erosive arthritis in SLE remains an open question, as several investigators have reported this [9, 46–50], whereas others have not [31, 45]. Kakumanu et al. reported a prevalence of 17% for anti-CCP positivity among 329 patients with SLE but that citrulline-dependent anti-CCP was mainly found in patients with erosive arthritis, which involved only 26 patients [9]. Pooled data from the present study indicate that 10 of 27 anti-CCP-positive patients (37%) had citrulline-dependent anti-CCP, which corresponds to 2.3% in the whole study population.

As previously mentioned, smoking affects the carbamylation process [16] and smoking is also strongly associated with anti-CCP-positive RA, especially in patients with the HLA-DRB1/shared epitope [52]. Thus, we anticipated that smoking would also be overrepresented among anti-CarP-positive and anti-CCP-positive patients with SLE. To our surprise, we did not detect such associations in this evaluation. In line with our findings, however, recent studies from Stockholm did not identify any correlation between smoking habits and anti-CCP in SLE [51], and anti-CarP was not significantly associated with smoking in patients with RA [11]. Of potential interest in relation to these results, the frequency of “ever” smokers in the discovery and replication cohorts was similar (approximately 45%), but was clearly lower than in two early RA cohorts (TIRA-2 and EIRA-1) from southern and central Sweden, where the frequency reached approximately 60% [52].

Evaluations with musculoskeletal US were performed in 32 patients with SLE (whereof 16 were anti-CCP positive) in the discovery cohort, showing significantly higher arthritis scores among anti-CCP-positive cases (Fig. 2a). Previous studies on musculoskeletal US in SLE with relevant comparators are scarce and a considerable strength in the present study is the matched control group (Table 2). Some studies have reported a higher number of affected tendons in SLE, compared to what we found [31, 53–55]. Interestingly, the effects of corticosteroids on B-mode/PD arthritis scores seemed to be limited (Fig. 2e).

We also investigated which RA-associated autoantibody-positive patients with SLE who simultaneously met the classification criteria for RA. This group could be referred to as “rhupus”, although there is no agreed definition for this overlap condition [25–27]. Although anti-citrullinated protein antibodies (ACPA) are indeed included in the 2010 ACR/EULAR criteria for RA, we used this most recent RA classification [43]. We found the risk of meeting RA criteria was higher given a positive anti-CCP test as compared to a positive anti-CarP antibody test (52% vs. 20%); when taking citrulline dependency into account for the anti-CCP test, this difference appeared to decrease. We considered using the 1987 ACR RA criteria (which does not include ACPA), but we were unable to retrieve sufficient data to determine fulfilment of this classification.

Apart from the statistically significant connection between a positive anti-CarP test and erosive arthritis in our study, the presence of anti-CarP was associated significantly with neurological involvement only in the discovery cohort, whereas a non-significant trend was found in the replication cohort. This may be due to the small number of patients with this clinical symptom. However, it is tempting to speculate that carbamylation may have an effect on neural tissue. Of relevance to this is the fact that supplementation of sodium cyanate to patients suffering from sickle cell anaemia disrupts the equilibrium between urea and cyanate in body fluids, resulting in increased carbamylation and severe polyneuropathy as side effects [56]. Furthermore, in animal models, cognitive impairment and loss of memory functions have been described as a result of increased carbamylation [57].

Whether or not anti-CarP antibodies can also be found in patients receiving sodium cyanate is unknown. It should be emphasized that both irreversible neuropathy and cognitive impairment are recorded as SLE damage accrual in the neuropsychiatric domain of the SLICC/ACR damage index [58]. Although renal function also affects the carbamylation process, we did not find any association between anti-CarP and renal involvement. The reason hereto is most likely that renal flares in well-controlled SLE are identified early and treated aggressively, resulting in a very small number of patients having end-stage renal disease [59]. In addition, carbamylation alone may not be sufficient for a break of tolerance against carbamylated proteins [60].

Although radiology data were at hand for all cases with a positive anti-CCP test, this was the case only for a minority of patients in the discovery cohort and not at all in the replication cohort, which is a limitation of our study. Furthermore, in the musculoskeletal US evaluations, it is likely that a larger number of evaluated patients with different autoantibody profiles would have enabled statistically significant and meaningful differences in anti-CarP and RF to be identified. Finally, anti-CCP, anti-CAP and RF were analysed with different assays in the two cohorts. The agreement with anti-CCP was fair, but we cannot exclude small methodological differences that might have influenced the results to some extent.

## Conclusions

In summary, we demonstrated that anti-CarP is more common than anti-CCP in well-characterised SLE in two European cohorts, whereas the overlap of these antibody specificities is limited. In the Swedish dataset we obtained some evidence that both autoantibodies are associated with erosive joint disease.
